# Third‐Generation Narrow‐Band Imaging Versus White‐Light Imaging for the Detection of Early Gastric Cancer: A Randomized Controlled Study

**DOI:** 10.1002/deo2.70288

**Published:** 2026-02-04

**Authors:** Yukihisa Fujinaga, Hitoshi Mori, Masayoshi Takami, Hiroyuki Masuda, Jun‐ichi Hanatani, Satoshi Iwai, Shohei Asada, Akihiko Shibamoto, Yuki Tsuji, Koh Kitagawa, Norihisa Nishimura, Shinya Sato, Kosuke Kaji, Tadashi Namisaki, Akira Mitoro, Hitoshi Yoshiji

**Affiliations:** ^1^ Department of Gastroenterology Nara Medical University Kashihara, Nara Japan; ^2^ Division of Endoscopy Nara Medical University Nara Japan

**Keywords:** detection rate of early gastric cancer, gastric cancer, gastrointestinal endoscopy, narrow‐band imaging, white‐light imaging

## Abstract

**Objectives:**

The endoscopic system EVIS X1 with improved image quality has been introduced into clinical practice. We examined whether third‐generation narrow‐band imaging (3G‐NBI) is more effective than white‐light imaging (WLI) for detecting early gastric cancer (EGC).

**Methods:**

Our study, performed at a single center, had a parallel‐group, open‐label, two‐arm, randomized, controlled design. Patients who had undergone endoscopic submucosal dissection for EGC were randomly assigned to a group undergoing 3G‐NBI after initial WLI (initial WLI group) or a group undergoing WLI after initial 3G‐NBI (initial 3G‐NBI group). The primary endpoint was the EGC detection rate of the two methods. The secondary endpoints were as follows: proportions of EGC detected and missed lesions, positive predictive value (PPV) for EGC diagnosis, and observation time for WLI and 3G‐NBI.

**Results:**

The EGC detection rate was 9.0% (17/188) in the initial WLI group and 8.5% (16/188) in the initial 3G‐NBI group. The missed lesion rate was 5.6% (1/18) in the initial WLI group and 0% (0/18) in the initial 3G‐NBI group. The PPV of the initial WLI group was 42.5% (17/40), whereas that of the secondary 3G‐NBI was 25% (1/4). The PPV of the initial 3G‐NBI group was 30.2% (16/53). No biopsies were performed during secondary WLI. The examination times were 274 ± 78.2 and 280 ± 82.9 s for WLI and 3G‐NBI, respectively.

**Conclusions:**

3G‐NBI was not superior to WLI in detecting EGC. This finding is likely due to improved WLI image quality.

**Abbreviations**
2G‐NBI: second‐generation narrow‐band imaging
3G‐NBI: third‐generation narrow‐band imaging
BAI‐MAC: brightness adjustment imaging with maintenance of contrast
BLI: blue laser imaging
EGC: early gastric cancer
ESD: endoscopic submucosal dissection
IEE: image‐enhanced endoscopy
LCI: linked color imaging
NBI: narrow‐band imaging
PPV: positive predictive value
TXI: texture and color enhancement imaging
WLI: white‐light imaging.

## 1 Introduction

Gastric cancer is one of the most prevalent oncologic diseases and the fifth leading cause of cancer‐related death worldwide [1]. The number of upper gastrointestinal endoscopies and the detection rate of early gastric cancers (EGCs) have increased with the increase in endoscopic screening. Although most EGCs can be cured with endoscopic resection, some are still missed during endoscopy. Reported lesion miss rates range from 4.6% to 25.8% [2, 3]. Meanwhile, advances in endoscopy—particularly image‐enhanced endoscopy (IEE)—have significantly improved the diagnostic accuracy for gastric epithelial neoplasms, outperforming white‐light imaging (WLI) [4, 5]. IEE includes modalities linked color imaging (LCI) (Fujifilm Medical Systems, Tokyo, Japan), blue laser imaging (BLI) (Fujifilm Medical Systems, Tokyo, Japan), narrow‐band imaging (NBI), and texture and color enhancement imaging (TXI) (Olympus Medical Systems, Tokyo, Japan). A comparative study demonstrated detection rates of 4.8% with WLI and 8.0% with LCI, with LCI significantly outperforming WLI in detecting superficial upper gastrointestinal tumors (*p* = 0.011) [6]. A comparative study reported detection rates of 4.8% with WLI and 8.0% with LCI, with LCI significantly outperforming WLI in detecting superficial upper gastrointestinal tumors (*p* = 0.011) [5]. Among IEE methods, NBI is particularly effective in detecting cancer, especially when used with magnifying endoscopy [7, 8]. However, NBI generates darker images than WLI, which potentially restricts visibility in the large gastric lumen; therefore, it may not have been optimal for screening purposes. A multicenter, prospective, and randomized controlled trial comparatively evaluated the detection rates of gastric neoplastic lesions between WLI and second‐generation NBI (2G‐NBI) and reported detection rates of 2.7% and 3.2%, respectively, indicating no significant differences between them (*p* = 0.412) [9].

The EVIS X1 endoscopic system (Olympus Medical Systems, Tokyo, Japan), introduced in 2020, is third‐generation technology. Its key features include: (1) high‐quality imaging, achieved with a high‐sensitivity complementary metal oxide semiconductor image sensor; (2) 4K image resolution, enabled by a high‐definition 4K UHD LCD monitor (OEV321UH) (Olympus Medical Systems); (3) extended depth of field, a function that synthesizes two images with different focal lengths to produce a single image in focus; and (4) Brightness Adjustment Imaging with Maintenance of Contrast (BAI‐MAC), which enhances brightness in darker distant areas while preserving foreground contrast. With these features, the EVIS X1 delivers brighter, higher‐quality images than previous systems. Kadota et al., in a phase II trial, evaluated the detection rates of gastric neoplastic lesions (EGC and adenoma) using WLI, 3G‐NBI, and TXI, and reported detection rates of 5.6%, 7.3%, and 5.0%, respectively [10]. Third‐generation NBI (3G‐NBI) evaluations are expected to be superior to WLI for EGC detection. This study evaluated the efficacy of 3G‐NBI compared to WLI in detecting EGC.

## 2 Materials and Methods

### 2.1 Study Design

The present study had a parallel‐group, open‐label, and two‐arm randomized controlled trial design and was carried out at the Department of Gastroenterology of Nara Medical University (Nara, Japan) between March 2022 and December 2024. The study protocol adhered to the ethical principles stipulated in the Declaration of Helsinki and was approved by the Institutional Review Board of Nara Medical University Hospital (approval no. 3189; date of approval: March 10, 2022). The present study has been included in the University Hospital Medical Information Network Clinical Trials Registry (UMIN000047156). This research was conducted according to the guidelines stipulated in the Helsinki Declaration of the World Medical Association. All the data were accessible by the authors, and the final version of the manuscript was reviewed by them and received their approval for journal submission. This study was reported in accordance with the CONSORT 2010 statement.

### 2.2 Participants

Metachronous gastric cancer recurrence is common, with a prevalence of 2.5%–14%, depending on follow‐up duration, and an annual incidence of 1%–3% [11, 12]. Given that these patients are at high risk, we included individuals who had undergone endoscopic treatment for EGC in our analysis.

The following patients were enrolled in this study: (1) those who had undergone endoscopic submucosal dissection (ESD) for EGC at Nara Medical University Hospital, and (2) those who were aged 20–85 years. Exclusion criteria were (1) history of gastrectomy or gastric tube reconstruction, (2) prior chemotherapy, (3) serious comorbidities, and (4) physician‐determined unsuitability. Written informed consent was obtained before the study.

### 2.3 Randomization

After enrollment, patients were assigned using simple randomization to either the initial WLI group (even‐month protocol) or the initial 3G‐NBI group (odd‐month protocol). Randomization was performed on a per‐patient basis. In the WLI group, the initial observation used WLI followed by 3G‐NBI; in the 3G‐NBI group, the initial observation used 3G‐NBI followed by WLI. Masking of group allocation was not conducted for the participating endoscopists.

### 2.4 Endoscopy

In the present study, we used the EVIS X1 endoscopy system, with the GIF‐XZ1200 (Olympus Medical Systems, Tokyo, Japan) upper gastrointestinal endoscope having optical magnification and OEV321UH 4K‐compatible LCD monitor. The BAI‐MAC setting was continuously maintained.

### 2.5 Endoscopic Diagnostic Criteria

**FIGURE 1:**
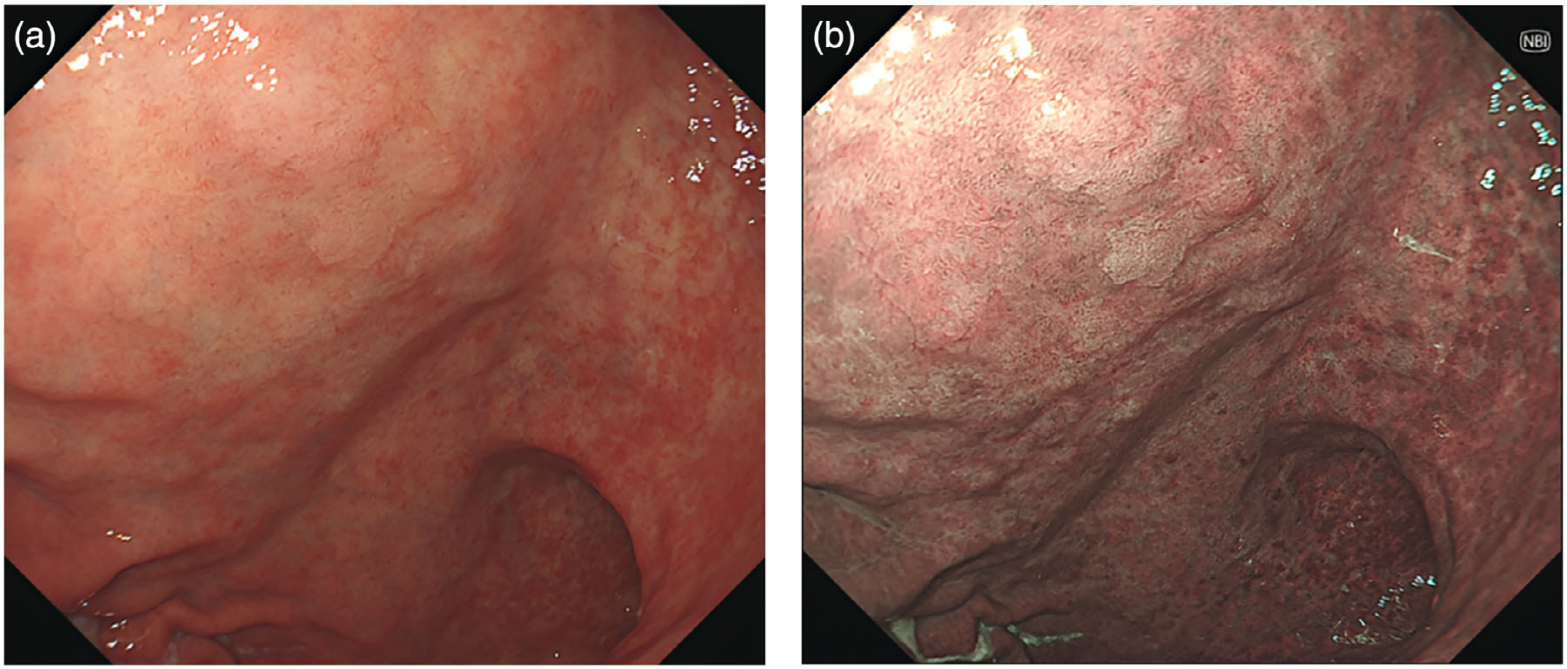
Representative endoscopic images of the identified target lesion. (a) White‐light imaging (WLI) demonstrating a discolored lesion with irregular margins. (b) Third‐generation narrow‐band imaging (3G‐NBI) revealing a brownish area with non‐uniform borders.

**FIGURE 2:**
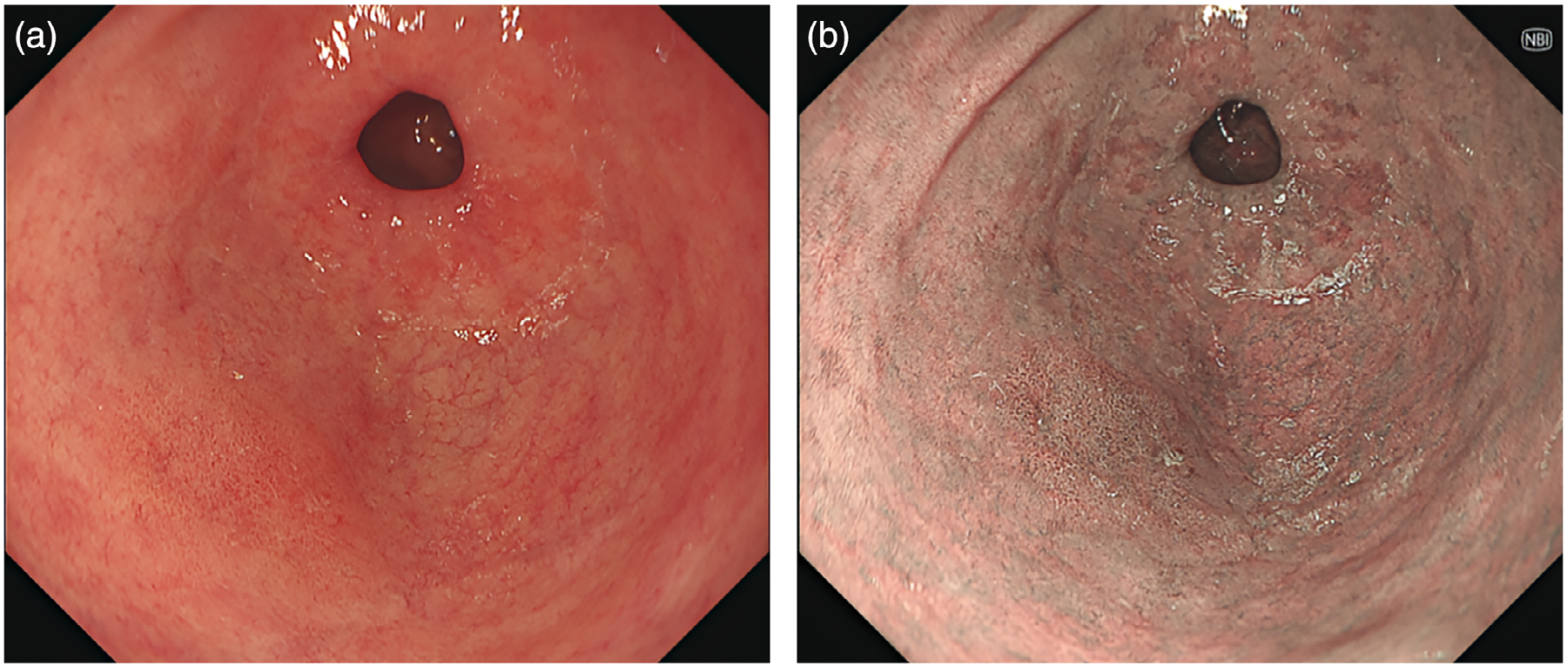
Representative endoscopic images of the identified target lesion. (a) White‐light imaging (WLI) revealing a reddish area with non‐uniform margins. (b) Third‐generation narrow‐band imaging (3G‐NBI) displaying a brownish area with non‐uniform margins.

EGC is defined as an epithelial malignant tumor originating in the gastric mucosa, with the invasion being confined to the mucosa or submucosa, not depending on the presence of lymph node metastases [13]. EGC suspected by non‐magnifying evaluation were designated as the target in our study. The latter were outlined as those exhibiting at least one of the following endoscopic features: (1) non‐uniform demarcation, (2) non‐uniform surface pattern, or (3) non‐uniform coloration. Lesions diagnosed at advanced stages were excluded from this category. The criteria for the target lesions were used for both WLI and 3G‐NBI (Figures 1 and 2).

*Helicobacter pylori* infection was confirmed by serum antibody, 13C‐urea breath, and rapid urease tests as well as histological examination. The *H. pylori* infection status after eradication treatment was determined based on the ^1^ ^3^C‐urea breath or stool antigen test findings.

Gastric mucosal atrophy was assessed based on the Kimura–Takemoto classification, which categorizes mucosal atrophy as the closed type, with atrophy not reaching the cardia, or the open type, with the atrophy extending beyond the cardia toward the greater curvature [14].

### 2.6 Examination Protocol

Initially, the entire stomach was evaluated using the assigned primary observation method, followed immediately by a re‐examination using the secondary observation method. During each non‐magnified assessment, if the lesions were suspected as EGCs, they were further evaluated using a combination of WLI and NBI. Thereafter, magnified NBI was used to assess the microvascular and microsurface structures; biopsies were obtained from irregular or absent pattern lesions within a demarcation line [13]. Observation time was defined as the duration of gastric examination, including lesion assessment and mucus/gas removal, excluding biopsy time.

### 2.7 Participating Endoscopists

All upper gastrointestinal endoscopic interventions were conducted by endoscopists accredited by the Japan Gastroenterological Endoscopy Society. Before the commencement of the study, all of the participating endoscopists underwent training in WLI and 3G‐NBI.

### 2.8 Pathological Diagnostics

Histopathological diagnostic examination was performed based on the examination findings of the biopsy or resected specimens obtained through the final endoscopic resection. Non‐malignant and malignant lesions were determined according to the modified Vienna classification [15]. Lesions belonging to category 4 (mucosal high‐grade neoplasia) or 5 (submucosal invasion by carcinoma) were classified as gastric cancer, whereas those referring to categories 1–3 were outlined as non‐cancerous.

### 2.9 Outcomes

The primary endpoint was the EGC detection rate during the initial examination with WLI and 3G‐NBI. The secondary endpoints were as follows: (1) the proportion of EGCs detected at initial examination among those detected during both initial and secondary examinations, (2) the proportion of missed EGC lesions at initial examination, (3) the PPV for the diagnosis of EGC during the initial examination, and (4) the observation time for WLI and 3G‐NBI during the initial examination. Missed EGCs were defined as the lesions that were detected during the second examination but were not identified during the initial assessment. The analyzed tumors were classified into subtypes based on their location, macroscopic appearance, and histological subtype.

### 2.10 Calculation of the Sample Size

The sample size was calculated to detect a statistically significant difference between 3G‐NBI and WLI in the primary endpoint of early gastric cancer (EGC) detection. A previous study using blue laser imaging (BLI)‐bright (Fujifilm Medical Systems, Tokyo, Japan), an IEE modality similar to WLI and NBI, reported a WLI detection rate of 4.0% (12/298), with a detection proportion of 50%, and a missed lesion rate of 50%. In contrast, BLI‐bright achieved 9.1% (27/298), 93.1%, and 6.9%, respectively [5]. BLI‐bright provides a brighter endoscopic view than conventional BLI. Our study targeted high‐risk patients and considered that EVIS X1 endoscopes offer improved image quality and brightness, likely resulting in a 3G‐NBI detection rate at least comparable to BLI‐bright. Accordingly, we assumed detection rates of 4% for WLI and 10% for 3G‐NBI, requiring 283 participants per group (566 in total) to achieve 80% power at a two‐sided significance level of 5%.

### 2.11 Statistical Analysis

The descriptive statistics for variables of age and observation time are shown as means ± standard deviations. Comparative analyses of quantitative data were done with the t‐test or Mann–Whitney U test. The assessments of differences in the proportions between the two groups were carried out with Fisher's exact test. Statistical significance was determined at *p* < 0.05. All statistical analyses were performed using EZR (version 1.68, Saitama Medical Center, Jichi Medical University, Saitama, Japan) [16], a graphical user interface for R (The R Foundation for Statistical Computing, Vienna, Austria), with the modifications allowing a broader command spectrum for biostatistics.

## 3 Results

**FIGURE 3:**
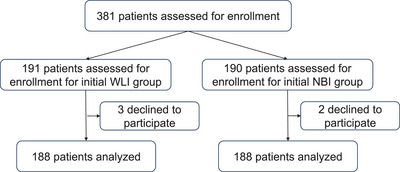
Flowchart showing patient enrollment. WLI, white‐light imaging; 3G‐NBI, third‐generation narrow‐band imaging.

Altogether, 381 cases were enrolled between April 2022 and December 2024 (Figure 3). The patients were randomly assigned to the initial WLI group (*n* = 191) or the initial 3G‐NBI group (*n* = 190). (Figure 3). After the randomization, five patients declined to participate. As a result, 188 patients in each group were examined. There were no adverse events.

The clinical features of the enrolled patients are reflected in Table 1. The initial WLI and 3G‐NBI groups did not differ significantly in sex, age, *H. pylori* infection status, or type of atrophic border, indicating that the clinical characteristics were well‐balanced.

**TABLE 1:** Clinical characteristics of patients.

Characteristic	Initial WLI group (*n* = 188)	Initial 3G‐NBI group (*n* = 188)	*p*‐Value
Sex			1
Male	148	147	
Female	40	41	
Age, years	75 ± 7.9	74 ± 9.0	0.507
*Helicobacter pylori*			0.8813
Present	1	1	
Eradicated	186	184	
Absent	1	3	
Atrophic border			0.7462
Closed type	20	23	
Open type	168	165	

Data are shown as means ± standard deviations.

Table 2 presents the EGC data obtained during the initial and secondary examinations, along with the biopsy results. In the initial WLI group, 17 EGC lesions were detected in 17 patients, whereas during the secondary 3G‐NBI, one lesion in one patient was detected. In the initial 3G‐NBI group, 16 EGCs were found in 16 patients during the initial 3G‐NBI, whereas none were observed during the secondary WLI. The detection rate of the initial WLI was 9.0% (17/188), with the proportion of detected EGCs composing 94.4% (17/18) and that of missed lesions being equal to 5.6% (1/18). The detection rate of the initial 3G‐NBI was 8.5% (16/188), with the proportion of detected EGC being equal to 100% (16/16) and that of missed EGC lesions being 0% (0/16). It could not be concluded that the detection rates differed significantly between the groups.

**TABLE 2:** Early gastric cancer (EGC) detected during initial and secondary examinations, biopsy results, and observation times in each group.

	Initial WLI group (*n* = 188)		Initial 3G‐NBI group (*n* = 188)		*p*‐Value
	Initial WLI	Secondary 3G‐NBI	Initial 3G‐NBI	Secondary WLI	
Patients with EGC, *n* (%)	17 (9.0)	1 (0.5)	16 (8.5)	0 (0)	1
Proportion of EGC detected, *n* (%)	17/18 (94.4)		16/16(100)		
Proportion of missed EGC lesions, *n* (%)	1/18 (5.6)		0/16 (0)		
Patients with target lesion, *n* (%)	38 (20.2)	4 (2.1)	46 (24.5)	0 (0)	
Target lesion, *n*	40	4	53	0	
EGC, *n* (%)	17 (42.5)	1 (25)	16 (30.2)	0 (0)	
Negative for neoplasia, *n* (%)	23 (57.5)	3 (75)	37 (69.8)	0 (0)	
Positive predictive value, %	42.5		30.2		
Observation time, seconds	274 ± 78.2	—	280 ± 82.9	—	0.7764

Descriptive statistics are displayed with number (%) or mean ± standard deviation.

In the initial WLI group, 40 lesions were biopsied, of which 17 were diagnosed as gastric cancers, resulting in a PPV of 42.5% for gastric cancer diagnosis. In the secondary 3G‐NBI examination, four lesions underwent biopsies, with one diagnosed as EGC, yielding a PPV of 25%. In the initial 3G‐NBI group, 53 lesions were biopsied, with 16 diagnosed as EGC, yielding a PPV of 30.2%. No lesion was biopsied during the secondary WLI. Due to the small number of cancer cases in our study, a PPV comparison was not performed. Regarding examination time, the initial WLI required 274 ± 78.2 s, whereas the initial 3G‐NBI required 280 ± 82.9 s.

Table 3 shows the clinicopathological characteristics of EGC detected during the initial and secondary evaluations. A total of 34 EGCs were identified through WLI and 3G‐NBI. ESD was performed for all the cases. It could not be proved if there were significant differences between the initial WLI and 3G‐NBI groups in tumor location or size. A statistical evaluation of macroscopic tumor type, tumor color, pathological classification, and invasion depth was not performed because some categories contained zero values.

**TABLE 3:** Clinicopathological characteristics of early gastric cancer (EGC) detected during initial and secondary examinations in each group.

	Initial WLI group (*n* = 188)		Initial 3G‐NBI group (*n* = 188)		*p*‐Value
	Initial WLI	Secondary 3G‐NBI	Initial 3G‐NBI	Secondary WLI	
EGC, *n*	17	1	16	0	
Location, *n*					0.8827
Upper third	2	0	4	0	
Middle third	9	0	7	0	
Lower third	6	1	5	0	
EGC size, mm					0.4994
<10	11	0	13	0	
10–20	6	1	3	0	
EGC color, n					—
Reddish	9	0	8	0	
Discolored	6	1	8	0	
Normal	2	0	0	0	
Pathologic type, *n*					—
Well‐differentiated	16	1	14	0	
Moderately differentiated	1	0	2	0	
Poorly differentiated	0	0	0	0	
Depth of invasion, *n*					—
T1a	17	1	13	0	
T1b	0	0	3	0	

The planned sample size was not achieved; therefore, an interim analysis was conducted at the end of the predefined study period, although this analysis had not been pre‐specified in the study protocol. According to the results during the study period, no clear superiority of 3G‐NBI over WLI in early cancer detection was observed. Extending the study period was considered unlikely to yield statistical significance; therefore, the study was concluded at the end of the study period despite not achieving the originally intended sample size.

## 4 Discussion

In the present research, the detection rates were 9.6% and 8.5% for WLI and 3G‐NBI, respectively. However, it could not be determined whether these parameters differed significantly between the WLI and 3G‐NBI groups. This finding may reflect the combined effects of improved brightness in both near and far fields achieved by BAI‐MAC and 4K‐equivalent image quality, which improve overall visual performance and allow WLI to achieve adequate visibility for EGC, thereby possibly narrowing the relative advantage conferred by the image‐enhancement properties of 3G‐NBI. Furthermore, endoscopists certified by the Japan Gastroenterological Endoscopy Society performed all upper gastrointestinal endoscopic examinations in this study. Therefore, the high level of expertise of the operators may have contributed to the relatively high EGC detection rate observed even with WLI. Conversely, the benefits of 3G‐NBI may become more evident when used by less‐experienced endoscopists. The detection rates could not be directly compared to those reported in previous studies due to the differences in the background characteristics of the analyzed patient populations.

In the present study, initial WLI achieved an EGC detection rate of 94.4% (17/18), whereas the rate of missed lesions was 5.6% (1/18). For the initial 3G‐NBI, the EGC detection rate was 100% (16/16), with no missed lesions (0/16). We could not infer that the detection rates differed significantly. In the current research, no significant differences in the detection rates were observed between the initial WLI and 3G‐NBI groups, and both modalities demonstrated low missed rates. These findings may be attributed not only to advancements in 3G‐NBI technology but also to improvements in the image quality of WLI itself.

The PPV for EGC detection was 42.5% for the initial WLI and 30.2% for the initial 3G‐NBI. The observation times were 274 and 280 s for the initial WLI and 3G‐NBI, respectively. NBI enhances vessel–mucosa contrast, and 3G‐NBI provides superior visualization compared to WLI during non‐magnified observation. Although the number of EGCs identified during the initial examination was comparable between the two groups, more lesions were detected with 3G‐NBI than with WLI during the pre‐magnification detection phase. As a result, 3G‐NBI showed a lower PPV and required a longer observation time than WLI.

With EVIS X1, WLI alone may achieve an acceptable detection level for EGC. Non‐magnifying 3G‐NBI may have a supportive role, considering the practical constraints of limited observation time.

The present study has several limitations. First, although statistical analyses were conducted, the interpretation of the results is not broad due to the insufficient sample size. Second, this was an open‐label study, and the endoscopists were not blinded. However, it was clinically impractical to change endoscopists in the first and second observations. Third, since this study targeted high‐risk patients for gastric cancer, the generalizability of the study findings may be limited.

## 5 Conclusions

In the present study, 3G‐NBI was not superior to WLI in determining the EGC rate. Although the study design lacked sufficient power to confirm non‐inferiority, the results for both modalities were generally comparable. This may be attributed to the improvements in imaging technology in both WLI and 3G‐NBI.

## Author Contributions

**Conceptualization**: Yukihisa Fujinaga. **Methodology**: Yukihisa Fujinaga, Norihisa Nishimura, Tadashi Namisaki, and Akira Mitoro. **Validation**: Akira Mitoro, Hitoshi Mori, Masayoshi Takami, Jun‐ichi Hanatani, and Hiroyuki Masuda. **Formal analysis**: Satoshi Iwai, Yuki Tsuji, Koh Kitagawa, Shinya Sato, and Kosuke Kaji. **Investigation**: Yukihisa Fujinaga. **Resources**: Yukihisa Fujinaga, Norihisa Nishimura, Shinya Sato, Kosuke Kaji, and Tadashi Namisaki. **Data curation**: Hitoshi Mori, Satoshi Iwai, Masayoshi Takami, Jun‐ichi Hanatani, Hiroyuki Masuda, Shohei Asada, Akihiko Shibamoto, and Yuki Tsuji. **Writing – original draft preparation**: Yukihisa Fujinaga. **Writing – review and editing**: Yukihisa Fujinaga, Akira Mitoro, and Koh Kitagawa. **Visualization**: Yukihisa Fujinaga, Shohei Asada, and Akihiko Shibamoto. **Supervision**: Akira Mitoro, Koh Kitagawa, and Hitoshi Yoshiji. **Project administration**: Hitoshi Yoshiji. All authors drafted the article and revised it critically to provide important intellectual content. All authors approved the final version to be published. All authors agreed to be accountable for all aspects of the work concerning the accuracy or integrity of any part of the work and ensuring that these issues were appropriately investigated and resolved. All authors have read and agreed on the published version of the manuscript.

## Conflicts of Interest

The authors declare no conflicts of interest.

## Funding

The authors have nothing to report.

## Ethics Statement

The present study was approved by the Institutional Review Board of Nara Medical University Hospital (approval no. 3189; date of approval: March 10, 2022).

## Clinical Trial Registration

This study was included in the University Hospital Medical Information Network Clinical Trials Registry (UMIN000047156).

## Consent

Informed consent was obtained from all the patients participating in the study.
